# Cyclic electron flow compensates loss of PGDH3 and concomitant stromal NADH reduction

**DOI:** 10.1038/s41598-024-80836-x

**Published:** 2024-11-26

**Authors:** Moritz Krämer, Nicolás E. Blanco, Jan-Ferdinand Penzler, Geoffry A. Davis, Benjamin Brandt, Dario Leister, Hans-Henning Kunz

**Affiliations:** 1 Plant Biochemistry, LMU Munich, Großhadernerstr. 2-4, 82152 Planegg-Martinsried, Germany; 2 Centre of Photosynthetic and Biochemical Studies (CEFOBI-CONICET-UNR), S2002LRK Rosario, Argentina; 3 Plant Molecular Biology, LMU Munich, Großhadernerstr. 2-4, 82152 Planegg-Martinsried, Germany

**Keywords:** Light responses, Non-photochemical quenching

## Abstract

In nature plants constantly experience changes in light intensities. Low illumination limits photosynthesis and growth. However, also high light intensities are a threat to plants as the photosynthetic machinery gets damaged when the incoming energy surpasses the capacity of photochemistry. One limitation of photochemistry is the constant resupply of stromal electron (e^-^) acceptors, mainly NADP. NADP is reduced at the acceptor-side of photosystem I. The resulting NADPH is utilized by the Calvin–Benson–Bassham cycle (CBBC) and the malate valve to ensure sufficient oxidized NADP ready to accept e^-^ from PSI. Lately, additional pathways, which function as stromal e^-^ sinks under abiotic stress conditions, were discovered. One such reaction in *Arabidopsis thaliana* is catalyzed by PHOSPHOGLYCERATE DEHYDROGENASE 3 (PGDH3), which diverts e^-^ from the CBBC into NADH. *pgdh3* loss-of-function mutants exhibit elevated non-photochemical quenching (NPQ) and fluctuating light susceptibility. To optimize plant photosynthesis in challenging environments knowledge on PGDH3’s metabolic integration is needed. We used the source of high NPQ in *pgdh3* as a starting point. Our study reveals that increased NPQ originates from high cyclic electron flow (CEF). Interestingly, PGDH3 function seems very important when the CEF-generator PROTON GRADIENT REGULATION5 (PGR5) is lost. Consequently, *pgr5pgdh3* double mutants are more sensitive to fluctuating light.

## Introduction

Plant photosynthesis is the foundation of higher life on earth. The pathway housed in the chloroplasts of leaf mesophyll cells, is separated into the directly light-dependent reactions, which include the two photosystems and the cytochrome *b*_6_*f* complex in the thylakoid membrane, and the light-independent reactions of the CBBC located in the chloroplast stroma. e^-^ carrier molecules such as plastoquinone (PQ), plastocyanin, ferredoxin, and finally NADP connect the individual components to guarantee maximum photosynthetic efficiency and plant growth. During linear e^-^ flow (LEF), e^-^ travel from PSII via the cytochrome *b*_6_*f* complex (cyt*b*_6_*f*) to PSI. At the PSI acceptor-side, e^-^ are transferred onto ferredoxin. Subsequently, most of the ferredoxin is oxidized by the enzyme ferredoxin reductase (FNR) yielding NADPH. During LEF, H^+^ are pumped from the stroma into the lumen, setting up a pH gradient, which drives the ATP-synthase. In addition, chloroplasts have the ability to bypass PSII and shuttle e^-^ exclusively between cyt*b*_6_*f* and PSI through a process called cyclic e^-^ flow (CEF). In most land plants, CEF has two routes. The first one, often referred to as the Antimycin A-sensitive path, utilizes PROTON GRADIENT REGULATION5 (PGR5)^1^. The other CEF route involves an independent mechanism, the multi-subunit NAD(P)H Dehydrogenase (NDH) Complex^2,3^. Both routes enable H^+^ pumping and therefore ATP production via the pH gradient of the trans-thylakoid proton motive force (pmf). The coexistence of LEF and CEF is critical for plant tolerance towards abiotic stress. For instance, cold temperature and fluctuating light both decrease NADP recycling in the stroma, which causes PSI acceptor-side limitation and concomitant PSI damage^4^. By redirecting e^-^ from reduced ferredoxin into the cyt*b*_6_*f*, CEF functions as a safety mechanism to avoid acceptor-side limitation thereby protecting PSI. Low lumenal pH has additional photoprotective implications^5^, a high lumen acidification triggers decreased cyt*b*_6_*f* redox turnover (photosynthetic control) and energy-dependent nonphotochemical quenching (NPQ ≈ qE) i.e., heat dissipation of absorbed light energy^6^.

Several additional mechanisms exist in the stroma to rebalance the redox poise by providing e^-^ sinks thereby minimizing damage to PSI (reviewed in Alric, J. & Johnson, X. (2017))^7^. The best-described mechanism is the chloroplast malate valve, which relies on two distinct malate dehydrogenases (MDH) and a malate shuttle. One MDH is specific for NADP(H) the other one for NAD(H)^8–11^. For a long time, the source of diurnal NADH in the stroma was unknown. Recently, the phosphorylated pathway of serine biosynthesis (PPSB) was identified as one NADH contributor. The committed reaction of PPSB is catalyzed by stromal PHOSPHOGLYCERATE DEHYDROGENASEs (PGDHs). The *Arabidopsis thaliana* genome encodes three isoforms of which only PGDH3 is expressed in the mesophyll where C_3_ photosynthesis takes place throughout the day^12^. *pgdh3* loss-of-function alleles exhibit elevated NPQ induction upon dark-to-light or high-to-low-light shifts and pronounced PSI acceptor-side limitation. Consequently, growth of *pgdh3* mutants is strongly affected under fluctuating light conditions. PGDH3 has a strong co-substrate preference for NAD over NADP. PGDH3 catalyzes the oxidation of 3-phosphoglycerate (3-PGA), the initial product of CO_2_ fixation provided by Ribulose-1,5-bisphosphate carboxylase/oxygenase (RuBisCO), to yield the serine precursor 3-phosphonooxypyruvate and NADH^13^. It was hypothesized that the withdrawal of 3-PGA from the CBBC aids to stabilize metabolism under strenuous conditions. PGDH3 provides an indirect route to replenish NADP and may avoid 3-PGA-dependent RuBisCO inhibition^14,15^. Recently, whole plant photosynthetic imaging under dynamic light conditions revealed a skewed relationship between LEF versus the lumen pH-dependent NPQ component qE in both *pgdh3* alleles compared to wild-type. This may reflect increased CEF in *pgdh3* mutants or other changes that alter the pmf^16^.

A deeper understanding of PGDH3’s physiological relevance could provide an avenue to optimize photosynthesis in challenging environments. To gain more insights into the functional integration of the PPSB into the stromal diurnal metabolism we set out to understand what mechanism(s) compensate the loss of PGDH3. Herein, we focused on identifying the source of elevated NPQ in *pgdh3* mutants and evaluated the crosstalk between photoprotective mechanisms and NADH turnover.

## Results

### High NPQ in *pgdh3* mutants is PsbS-dependent

**Fig. 1:**
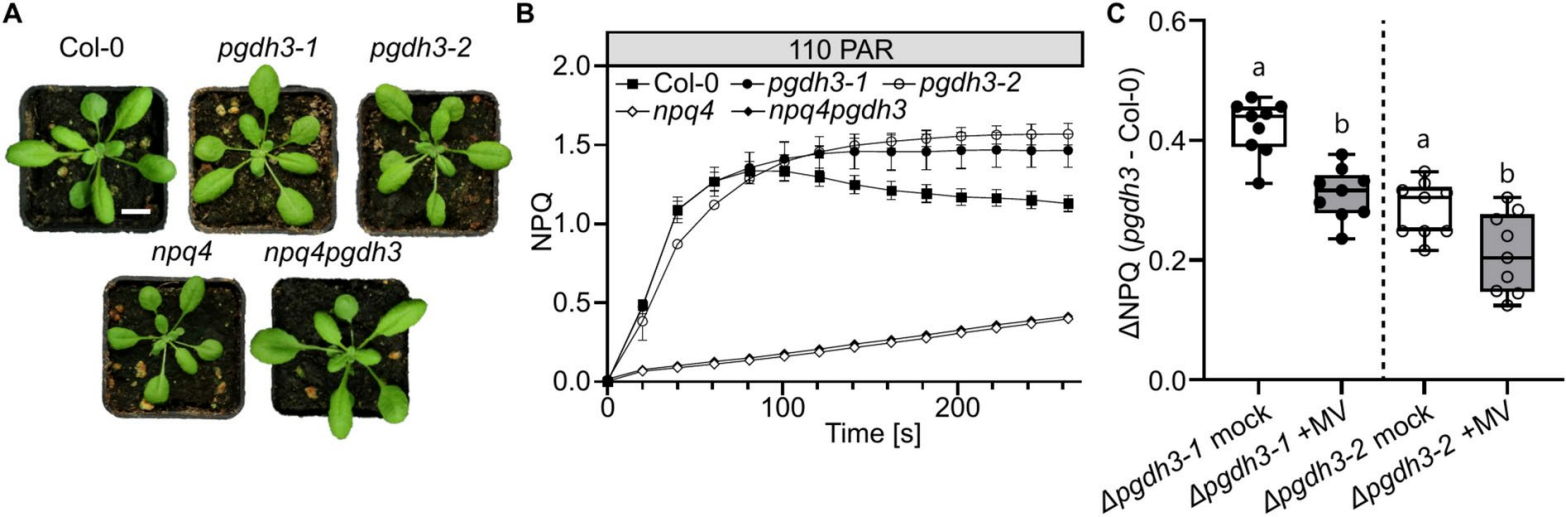
Elevated Non-Photochemical-Quenching (NPQ) in *PGDH3*-deficient mutants originates from high qE. **A** 3-week-old plants grown under standard long-day conditions. Scale bar = 1 cm. **B** NPQ induction curve measured at 110 PAR for Col-0 (filled squares), *pgdh3-1* (filled circle), *pgdh3*-2 (empty circle), *npq4* (empty diamond), and *npq4pgdh3* (filled diamond). Mean, ± SD, N = 8–9. **C** ΔNPQ (*pgdh3* NPQ – Col-0 mean NPQ) with and without methyl viologen (MV) treatment. 200 µM MV was sprayed onto the leaves right before dark adaptation for measurements. Mean, Min to Max, N = 9, P < 0.05.

NPQ consists of several components (qE, qZ, qI, qT, and qH)^17^, with the PsbS-dependent qE-component being the fastest and major contributor^18,19^. To pinpoint the source of high NPQ in *pgdh3* plants *pgdh3npq4* double mutants were generated. Under ambient growth conditions the general appearance of all mutants is indistinguishable from wild-type plants (Fig. 1A). In an induction curve at a light intensity of 110 µmol photons m^−2^ s^−1^ (PAR), *pgdh3* plants exhibit a characteristic increase in transient and steady-state NPQ compared to wild-type controls (Fig. 1B). The elevated effect depends on the function of PsbS, since *pgdh3npq4* plants show no significant change from *npq4* single mutants. It follows that high NPQ in *pgdh3* plants consists primarily of high qE.

Next up, we tested if the PSI acceptor-side limitation in *pgdh3* is linked to the elevated NPQ. Therefore, we sprayed wild-type and mutant plants with methyl viologen (MV) an artificial electron acceptor which competes with ferredoxin for e^-^ from PSI^20^. In the presence of MV, NPQ values in *pgdh3* are significantly closer to wild-type control than in the mock-treated samples (Fig. 1C).

### Transcriptomics and immunoblots show that high NPQ in *pgdh3* mutants does not result from alterations in photosynthetic genes and proteins

**Fig. 2:**
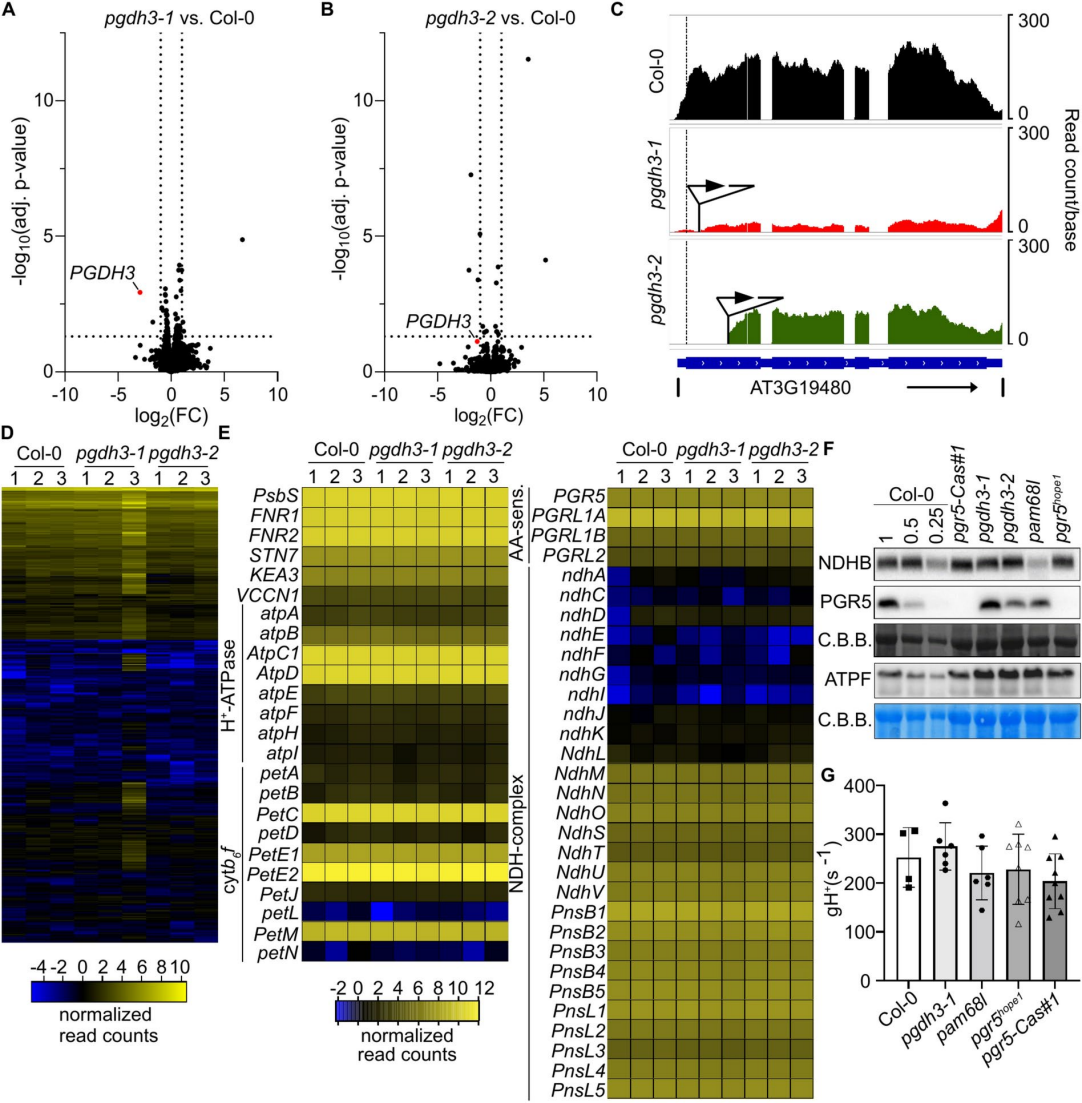
Transcriptomic and immunoblot analysis of mRNA and protein levels in WT and *pgdh3* mutant plants. **A-B** Volcano plots of *pgdh3-1* (A) or *pgdh3-2* (B) vs Col-0 RNAseq experiments. Dotted lines depict a 0.05 adjusted *p*-value or log_2_ fold change (FC) of |1| cutoff, respectively. The red dot represents *PGDH3* transcripts. **C** Read coverage of the PGDH3 (AT3G19480) locus of selected bio-replicates of WT (black), *pgdh3-1* (red), or *pgdh3-2* (green) after read mapping. The gene structure is given under the coverage plots in blue. The dashed line indicates the start codon of the gene while triangles show the location of the respective T-DNA insertions. **D** Heatmap showing normalized read counts of the 1000 most diverse genes (by standard deviation) of all three bio-replicates of WT, *pgdh3-1,* and *pgdh3-2* plants. Rows are clustered hierarchically. **E** Non-clustered heat map of normalized read counts for selected genes which could influence ΔpH and NPQ, respectively, in Col-0, *pgdh3-1,* and *pdgh3-2* plants. **F** Immunoblots (normalized to 15 µg total protein) of NDHB, PGR5, and ATPF with Coomassie brilliant blue staining of RbcL and Col-0 dilution (1; 0.5; 0.25 × total protein) as loading controls. **G** Thylakoid proton conductivity gH^+^(s^-1^) under standard growth conditions in 3-week-old plants. Col-0 (filled squares), *pgdh3-1* (filled circle), *pam68l* (filled hexagon), *pgr5*^*hope1*^ (empty triangle), *pgr5-Cas#1* (filled triangle) Mean, ± SD, N > 4.

Since elevated NPQ in *pgdh3* is dependent on PsbS function, two possible scenarios in the mutant plants exist: a) a more acidic thylakoid lumen resulting in stronger PsbS activation or b) an upregulation of *PsbS* transcript and protein amounts since *PsbS* overexpression triggers NPQ in plants^21^. To check if this is the case in *pgdh3* plants and to identify other putative compensatory mechanisms, we performed an RNAseq experiment on plants grown in ambient light conditions. All our quality checks suggested high data reproducibility within our mutant panel (Suppl. File 1). While, as expected, *PGDH3* was among the lowest expressed genes in *pgdh3-1* and exhibited a truncated transcript with slightly lower abundance for *pgdh3-2* (Fig. 2A-B), only a small number of significantly differentially expressed genes (DEGs) was identified under ambient growth conditions when comparing both mutant lines with WT plants (Fig. 2C, and Suppl. File 2). However, these DEGs were not consistently changed between the two alleles and thus are likely unrelated to the loss of *PGDH3* (Fig. 2D, Suppl. Fig. S1A, and Suppl. File 2). This impression was further confounded by plotting gene transcripts with known impact on thylakoid lumen H^+^ level and NPQ, such *PsbS*, *KEA3*, the cyt*b*_6_*f* complex, the two CEF pathway components, and the ATP synthase^22^. For genes of these complexes, no significant mRNA level differences were found between the genotypes (Fig. 2E). It follows that high NPQ in *pgdh3* plants is not due to transcriptional changes of *PsbS* or other compensatory gene networks related to photosynthesis.

Since some Arabidopsis genes have no proportional relationship between mRNA abundance and protein levels^23^, we probed protein complexes with H^+^ pumping activity and therefore well-known effects on the thylakoid lumenal pH i.e., the NDH complex, the PGR5/PGRL1 complex, and the multi-subunit ATP synthase by immunoblotting. For the two CEF routes, we employed previously established loss-of-function alleles as controls, namely *PHOTOSYNTHESIS AFFECTED MUTANT68-LIKE* deficient *pam68l-1*, which is devoid of NDH, and two independent *pgr5* mutants. The original *pgr5-1* line carries a second site mutant in *PSI PHOTOPROTECTION1/ CONSERVED ONLY IN THE GREEN LINEAGE20* (*PTP1/CGL20*, AT2G17240)^24,25^, which alters the NDH complex stability. Therefore, we utilized two recently published independent alleles: *pgr5*^*hope1*^^26^ and *pgr5*-Cas#1^27^, both of which have been confirmed by whole-genome re-sequencing.

While the controls confirmed absence or lower levels of PGR5 and NDH in respective loss-of-function mutants, none of the proteins revealed clear abundance changes in *pgdh3*. The ATP synthase was unchanged regardless of the genotype (Fig. 2F). Overall, the immunoblots are in line with the unchanged transcript amounts observed in the RNAseq experiment. Lastly, we determined the thylakoid membrane proton conductivity (gH^+^) as a proxy for ATPase activity^28^. Confirming the ATP synthase immunoblots, no significant changes from wild-type controls were observed in any mutant allele (Fig. 2G, ECSt Suppl. Figure 1B).

In summary, elevated NPQ in *pgdh3* mutants is not caused by alterations in LEF, CEF, and ATP synthase components. Therefore, it seems that in the *pgdh3* plants, compensatory mechanisms that yield lower lumenal pH are merely increased in their activities but not their abundance.

### The loss of PGDH3 results in higher CEF rates

**Fig. 3:**
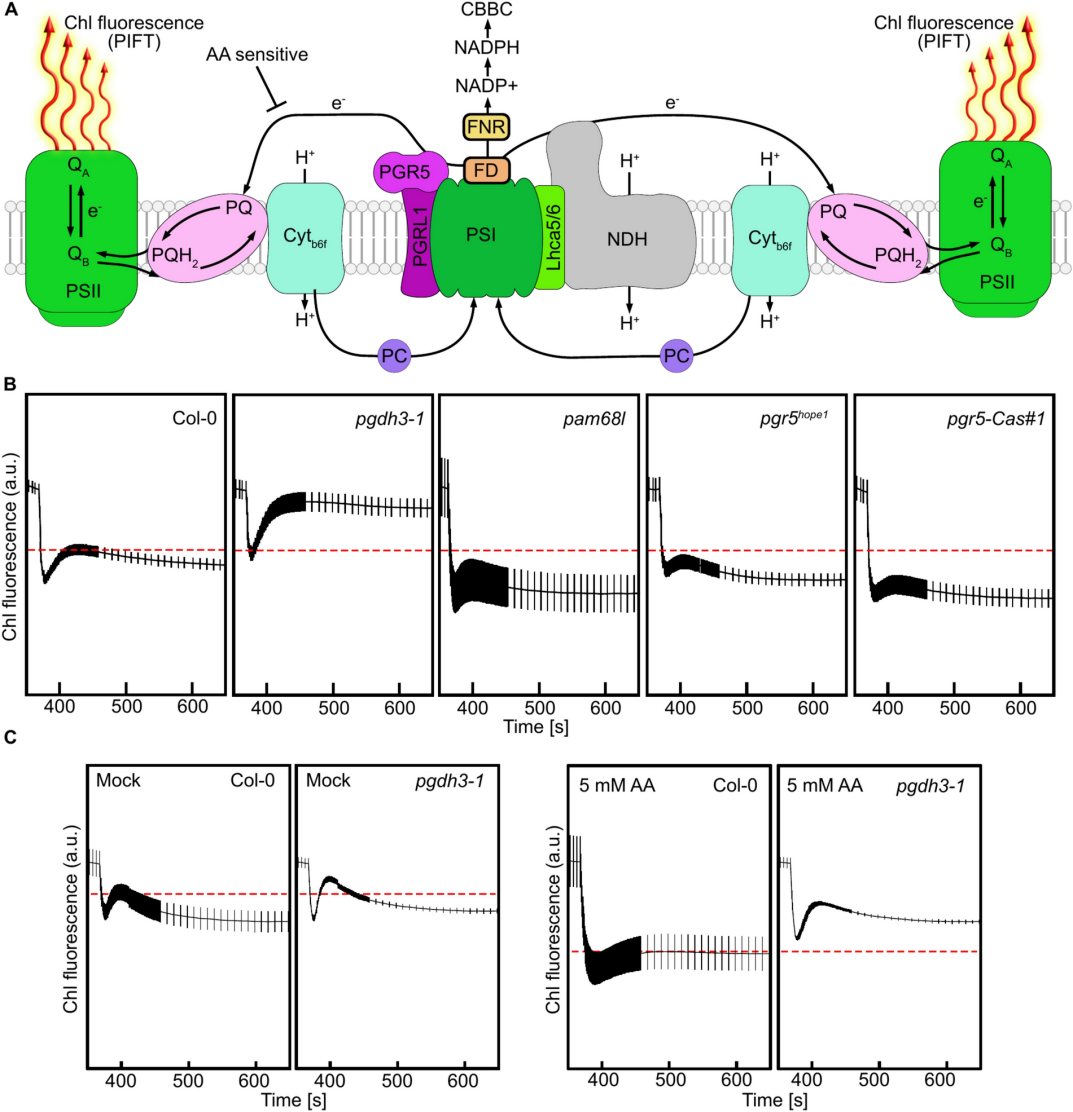
The lack of PGDH3 results in high cyclic electron flow (CEF). **A** CEF triggering the Post-Illumination Chlorophyll Fluorescence Transient (PIFT) signal at PSI upon darkness (modified from Strand, D. D., Fisher, N., & Kramer, D. M. (2017))^29^. Linear electron flow (LEF) directs electrons (e^-^) from photosystem II (PSII) through plastoquinone (PQ) reduction to plastoquinol (PQH_2_) via the cytochrome *b*_6_*f* complex (Cyt*b*_6_*f*) and subsequent reduction of plastocyanin (PC) to photosystem I (PSI). Cyt*b*_6_*f* transfers the two protons (H^+^) from PQH_2_ to the thylakoid lumen generating a proton motive force and a ΔpH. Following LEF e^-^ can be transferred to ferredoxin (FD) and then onto NADPH via ferredoxin NADP reductase (FNR), which fuels Calvin Benson Bassham cycle (CBBC) etc. Alternatively, e^-^ can be recycled to the PQ-Pool via two CEF routes. In the AA-sensitive route e^-^ are transferred from FD by the proton gradient regulation 5 (PGR5) PGR5-like photosynthetic phenotype 1 (PGRL1) complex to PQ. The second route utilizes the NADH dehydrogenase-like (NDH) complex bound via light-harvesting complex I subunits 5 and 6 Lhca5/6 to PSI forming the PSI-NDH supercomplex. This supercomplex recycles e^-^ from FD to the PQ-Pool while pumping H^+^ from the stroma to the lumen. The PIFT constitutes PSII Chl fluorescence that can be measured when the light is turned off as it comes to an immediate fluorescence drop followed by a fluorescence increase caused by e^-^ transferred via either route of CEF reducing PQ to PQH_2_. As PSII enters an inactive state some e^-^ are transferred to the PSII Q_B_ site resulting in Q_A_ equilibration and a detectable Chl fluorescence rise. **B** PIFT of 3-week-old plants after illumination at 56 PAR of the genotypes Col-0, *pgdh3-1*, *pam68l*, *pgr5*^*hope1*^, and *pgr5-Cas#1*. The dotted red line indicates the Col-0 F_0_ peak in PIFT. Mean, ± SEM, N = 9. **C** PIFT after infiltration (before dark adaptation) with Antimycin-A or water (mock), respectively. The dotted red line indicates the Col-0 F_0_ peak in PIFT. Col-0, and *pgdh3-1*. Mean, ± SEM, N = 3.

Since the ATP synthase activity was unchanged (Fig. 2G), we probed CEF in our mutant panel. Here, we quantified the post-illumination fluorescence signal, a transient rise in chlorophyll fluorescence subsequent to an initial fluorescence drop (often referred to as PIFT or PIFR)^29,30^, upon switching off the actinic light on illuminated leaves in photosynthetic steady state. Under such conditions, stromal e^-^ are retransferred to the plastoquinone (PQ) pool via both CEF pathways to varying degrees (Fig. 3A). Subsequently, PQH_2_ equilibrates with the PSII quinones (Q_B_, Q_A_) and a detectable chlorophyll a fluorescence emerges^29,31^.

Interestingly, *pgdh3* alleles exhibit a much stronger PIFT signal suggesting higher CEF rates compared to wild-type plants (Fig. 3B, Suppl. Figure 2A). We also plottet Y(I) versus Y(II), another indicator of changes in CEF, which confirmed increased CEF in *pgdh3* compared to wild-type controls (Suppl. Figure 2B). As expected, in *pam68l-1* mutants, defective in NDH, the fluorescence rise signal was almost absent. Both *pgr5* alleles also had lower PIFT than wild-type, confirming that the PIFT signal is in part also fed by the Antimycin A-sensitive route (Fig. 3B). Treating *pgdh3* plants with 5 mM Antimycin A decreased but did not fully abolish the PIFT (Fig. 3C).

**Fig. 4:**
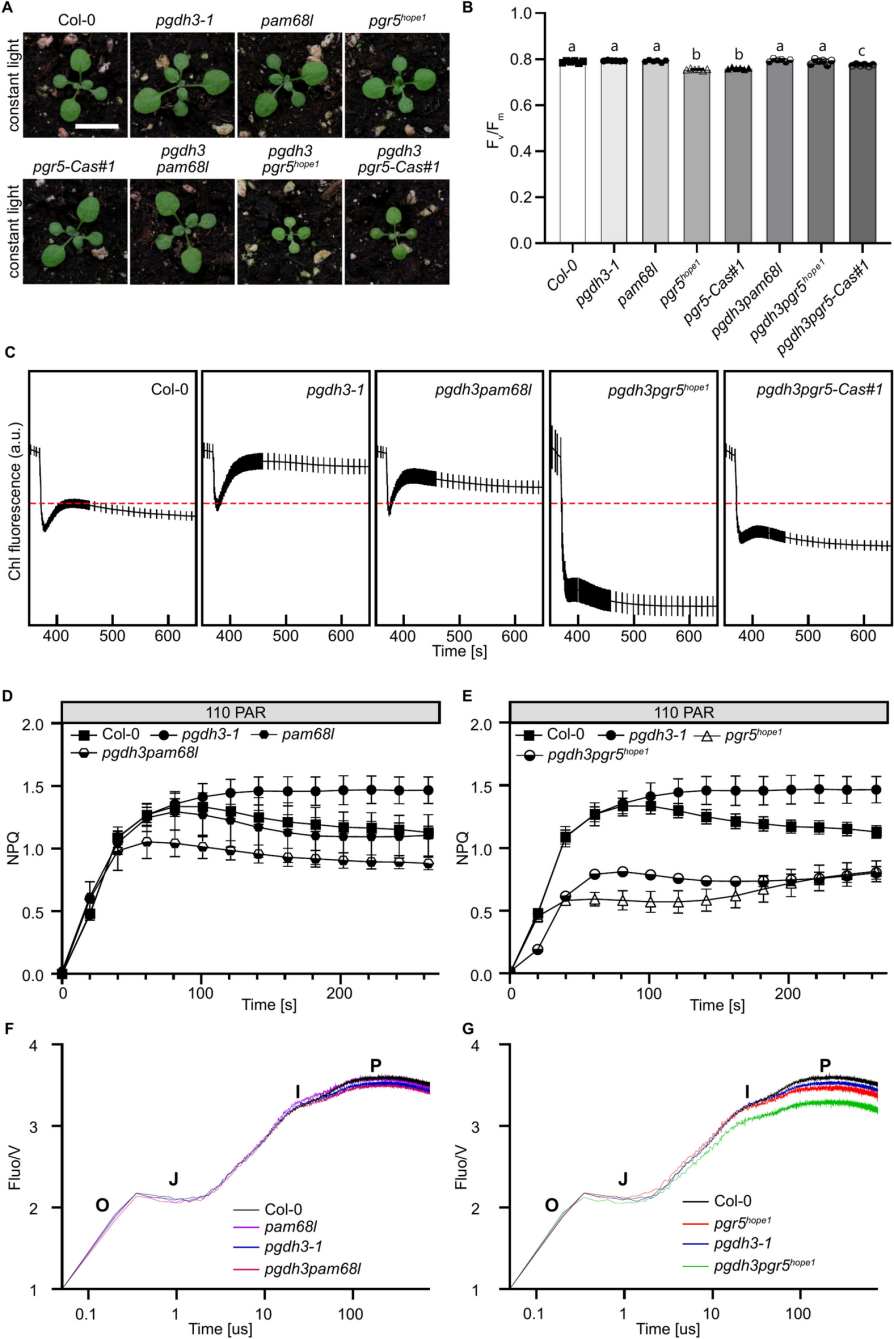
Characterization of higher order mutants of *pgdh3* with either *pgr5* or *pam68lpam68l. A* 14-day-old plants grown under standard growth conditions. While *pgdh3pam68l* appears similar in size and color to Col-0 and single mutants, *pgdh3pgr5*^*hope1*^*,* and *pgdh3pgr5-Cas#1* lines are not only smaller but also pale green. **B** F_v_/F_m_ determination for single and double mutant lines. Mean, ± SD, N = 9, P < 0.05. **C** PIFT measurement of 3-week-old plants of the genotypes Col-0, *pgdh3-1*, *pgdh3pam68lpam68l*, *pgdh3pgr5*^*hope1*^, and *pgdh3pgr5-Cas#1*. The dotted red line indicates the Col-0 F_0_ peak in PIFT. Mean, ± SEM, N = 9. **D**, **E** Non-Photochemical-Quenching (NPQ) induction curve measured at 110 PAR for Col-0 (filled squares), *pgdh3*-1 (filled circle), *pam68l* (filled hexagon), *pgr5*^*hope1*^ (empty triangle), *pgdh3pam68l* (half-filled hexagon) and *pgdh3pgr5*^*hope1*^ (half-filled circle). Mean, ± SD, N = 6–9. **F**, **G** OJIP curves were taken with the standard Dual-PAM protocol for the different genotypes. Col-0 (black), *pgdh3*-1 (blue), *pam68l* (purple), *pgr5*^*hope1*^ (red), *pgdh3pam68l* (pink) and *pgdh3pgr5*^*hope1*^ (green). MEAN, N = 6.

Since the PIFT signal does not allow to evaluate the relative contribution of NDH- and PGR5-permitted CEF in *pgdh3* loss of function lines, we designed higher order mutants (Suppl. Figure 3). While the single and *pgdh3pam68l* mutant plants were indistinguishable from wild-type, the *pgdh3pgr5* alleles appeared slightly smaller with paler leaves and sometimes visible leaf veins (Fig. 4A). However, chlorophyll contents in double mutants did not reveal any significant decrease compared to previously observed slight reductions in *pgr5* and *pgdh3* single mutants (Suppl. Figure 4A)^13,32^. The maximum quantum yield of PSII, *F*_v_/*F*_m_, remained largely unchanged (Fig. 4B). Also, a *pgr5pam68l* mutant was isolated. As previously described, mutants defective in both CEF pathways grow very slowly, display pale leaves with clearly visible veins, and an overall sick appearance^25,33^. All these hallmarks were noticeable in *pgr5pam68l* plants (Suppl. Figure 5).

In *pgdh3pam68l* plants CEF was lower than in *pgdh3* loss of function lines (Fig. 4C). Whereas in *pgr5pgdh3* individuals the PIFT signal was equally low as in *pgr5* single mutants. Next up, the transient NPQ was determined. *pgdh3* plants exhibited characteristically high NPQ, *pam68l* single mutants showed mildly, statistically not significant, lower than wild-type transient NPQ (Fig. 4D). However, *pgdh3pam68l* double mutant plants had significantly lower NPQ than both respective single mutants and the wild-type controls (Fig. 4D). The newly isolated *pgr5pgdh3* double mutants had lower NPQ than *pgdh3* single mutants and wild-type plants but were statistically indistinguishable from either *pgr5* single mutant allele with regards to their transient NPQ (Fig. 4E, Suppl. Figure 4B). Altogether, the lack of PGDH3 and concomitant NADH production is compensated by increased CEF rates with both CEF pathways contributing to different degrees.

To gain further insights, fast chlorophyll *a* fluorescence induction (OJIP) transients on dark-adapted plants were recorded. No differences between genotypes emerged in the early O-J (photochemical) phase, which gives insights into PSII and PQ e^-^-transfer. In contrast, during the later amplitudes i.e., the J-I-P (thermal) phase indicative of the PSI electron acceptor pool (ferredoxin, NADP, FNR levels, and activity, respectively)^34^, the mutants diverged from the wild-type. *pgdh3pam68l* and its respective single mutants were no different from each other but all showed a slight J-I-P fluorescence decrease compared to the wild-type controls (Fig. 4F). This drop was more pronounced in *pgr5* alleles (Fig. 4G). However, *pgdh3pgr5* double mutants showed a substantial, additive decrease in the J-I-P phase from wild-type and both respective single mutant lines. This finding suggests that during the induction of photosynthesis, and possibly during low to high light shifts, PSI activity and the prevention of severe PSI acceptor-side limitation in *pgdh3* mutants hinges more on PGR5 activity to produce high CEF rates than it does on the NDH-pathway for CEF.

### PGDH3 activity remedies PSI acceptor-side limitation in *pgr5* mutants and vice versa

**Fig. 5:**
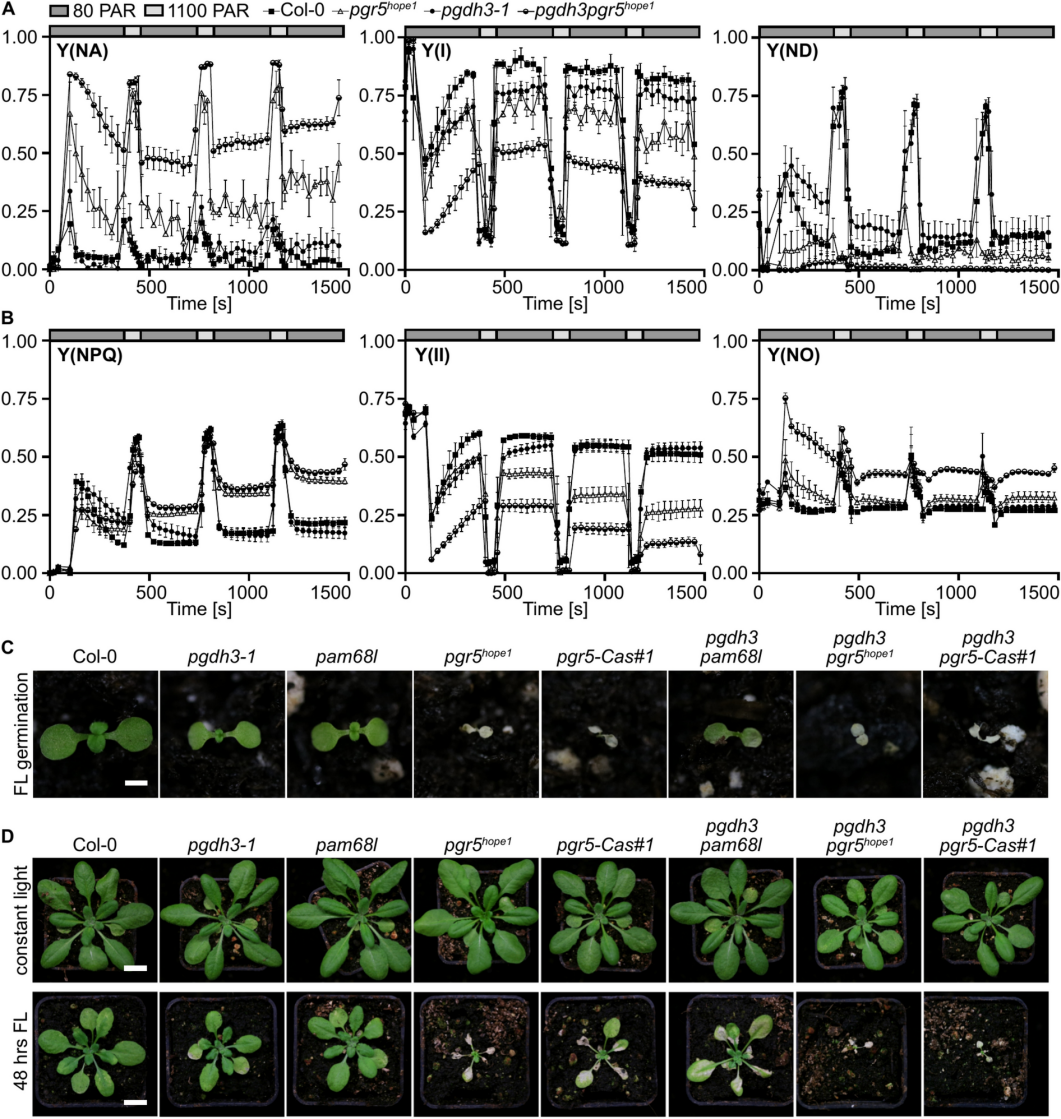
Simultaneous loss of PGDH3 and PGR5-dependent CEF strongly affects the PSI acceptor-side and decreases higher loss-of-function mutant survival under fluctuating light treatments. **A, B** Fluctuating light measurements (5 min low light (80 PAR) followed by 1 min high light (1100 PAR)) were carried out on 3-week-old plants to determine PSI acceptor-side limitation (Y(NA)), PSI donor site limitation (Y(ND)), photochemical quantum yield of PSI (Y(I)), quantum yield of regulated energy dissipation (Y(NPQ)), quantum yield of nonregulated energy dissipation (Y(NO)), and effective PSII quantum yield (Y(II)). Genotypes: Col-0 (filled squares), *pgdh3-1* (filled circle), *pgr5*^*hope1*^ (empty triangle), and *pgdh3pgr5*^*hope1*^ (half-filled circle). Mean, ± SEM, N = 3. **C** 12-days-old seedlings germinated and grown under fluctuating light conditions (5 min at 50 PAR low light, 1 min at 500 PAR high light) for the genotypes Col-0, *pgdh3-1*, *pam68l*, *pgr5*^*hope1*^, and *pgr5-Cas#1, pgdh3pam68l*, *pgdh3pgr5*^*hope1*^, and *pgdh3pgr5-Cas#1.* Scale bar = 1 cm. **D** 3-week-old plants of the aforementioned genotypes were subjected to control and fluctuating light conditions and given one week of recovery afterward. Scale bar = 1 cm.

One hallmark of PGR5-deficiency is a highly increased PSI acceptor-side limitation (Y(NA))^35^. The loss of *PGDH3* also results in increased PSI acceptor-side limitation albeit to a much lower degree^13^. Fluctuating growth light is known to exacerbate stress on the electron transfer at the stromal PSI site^36^. Therefore, we subjected adult plants of our mutant panel to a short-term light fluctuation experiment (4 cycles of 5 min at 80 PAR, followed by 1 min at 1100 PAR) and recorded several PSI- (Fig. 5A, Suppl. Figure 6–7) and PSII-related parameters (Fig. 5B, Suppl. Figure 8). *PGDH3-* and *PGR5*-deficient single mutants replicated their previously reported Y(NA) behaviors^13,37^. Changes in the slope of Y(I) vs Y(II) again indicated increased CEF in *pgdh3* albeit this effect tapered off over the course of the experiment (Suppl. Figure 6). Strikingly, *pgdh3pgr5* alleles showed additive effects i.e., an extremely high PSI acceptor-side limitation during low and high-light phases. This behavior was accompanied by decreased quantum yield of PSI Y(I) and PSI donor side Y(ND) (Fig. 5A). It follows that during high light cycles, PSI in *pgdh3pgr5* double mutants was almost fully oxidized, suggesting a failure to buildup ΔpH-dependent photosynthetic control. Interestingly, *pgr5* single and *pgdh3pgr5* double mutants exhibited high Y(NPQ) and Y(NO) at the cost of Y(II) during low light phases (Fig. 5B).

A similar behavior in Y(NA) was not noticeable in NDH-defective *pam68l* and *pgdh3pam68l* double mutants. Here, only *pam68l* single mutants displayed mild increases in Y(I) and Y(ND) (Suppl. Figure 7B).

The extreme Y(NA) behavior in *pgdh3pgr5*, led us to test the growth behavior of the mutant panel in response to an extended fluctuating light period. Initially, we replicated the well-established germination experiment under constant versus fluctuating growth light (5 min at 80 PAR, followed by 1 min at 1100 PAR)^38^. Under these conditions, single as well as *pgdh3pgr5* alleles failed to establish (Fig. 5C).

Therefore, a second experiment was set up. Here, plants were grown into a more adult stage before the initiation of the flowering stage. At the age of two-weeks, the plants were subjected to the same fluctuating light treatment as during the germination experiment or kept at constant long-day light conditions (Fig. 5D).

Wild-types tolerated 48 h of fluctuating light treatment showing only a mild growth reduction compared to constant light control plants. In *pgdh3* and *pam68l* plants older leaves displayed early signs of tissue damage when exposed to light stress. This behavior was more pronounced in *pgdh3pam68l* double mutants. Both *pgr5* loss-of-function alleles were strongly photodamaged by the changing illumination and displayed extensive lesions. The Col-0 allele *pgr5*-Cas#1 was less severely damaged than the *pgr5*^*hope1*^ mutant, which was isolated in the trichome-free leaf gl-1 background^39^. Interestingly, while *pgr5* single mutants were able to grow back and survive once the fluctuating light treatment was stopped this was not possible in *pgr5pgdh3* double mutants.

In summary, the spectroscopy and growth study under fluctuating light conditions revealed that PGDH3 function and its linked activity to produce NADH are critical to delay photodamage and acceptor-side limitation of PSI. This is especially important when the PGR5-dependent CEF pathway is inactive e.g., in *pgr5* loss-of-function mutants.

## Discussion

PSI acceptor-side limitation presents a highly dangerous situation for plant survival since PSI turnover takes much longer (days) than for PSII (minutes)^40,41^. CEF and several other mechanisms located in the stroma prevent a toxic buildup of e^-^ at the PSI acceptor-side ^7,42^. Reactions that replenish NADP, the main e^-^ acceptor of ferredoxin^43^, contribute to different degrees in delaying PSI photodamage^44^. Historically, research on stromal NADP(H) has dominated the literature. However, NADP is produced from NAD in the stroma^45^. Hence an NADP(H) and an NAD(H) pool must coexist in the chloroplast^4^. The discovery of a NAD(H)-dependent MDH has fueled the idea that NAD(H) also plays a role in adjusting the stromal redox poise^9–11^. Nevertheless, reactions to produce stromal NADH during the light phase remained unknown for a long time^4^. Recently, PGDH3, the committing step of the PPSB, was shown to yield NADH during the day. *pgdh3* loss-of-function plants suffer from a limited stromal electron sink. This results in elevated PSI acceptor-side limitation, increased NPQ, and fluctuating light sensitivity^13,16^. A detailed understanding of the PPSB may provide a useful engineering target to optimize photosynthesis in challenging environments. Hence, we set out to pinpoint the source of high NPQ in *pgdh3* loss-of-function mutants and understand the integration of PPSB into the chloroplast metabolic network.

High NPQ in *pgdh3* lines consists primarily of qE i.e., a lower lumenal pH (Fig. 1B). We found no evidence that a transcriptional or protein response is activated in *pgdh3* mutants under ambient light conditions (Fig. 2). Therefore, activity adjustments of preexisting bypass reactions are sufficient to buffer the defects of PGDH3. This may be different under more adverse stress conditions such as continuous fluctuating light stress when *pgdh3* mutants exhibit leaf damage ^13^, indicating that compensatory mechanisms have reached their limits. Indeed, the buffering capacity of the plant proteome towards changes in photosynthesis is quite high. For instance, thylakoid ion flux-deficient mutants, which show NPQ changes under ambient conditions, also do not reveal a strong transcriptional response^46^.

**Fig. 6:**
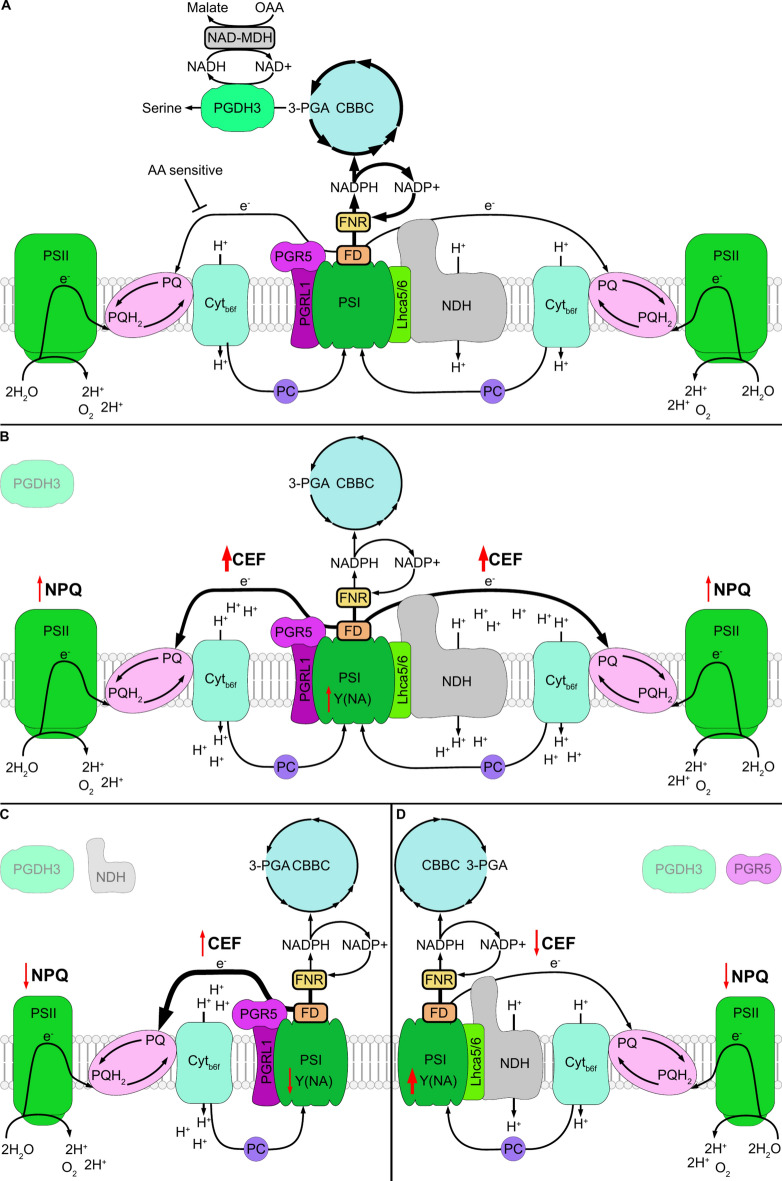
Mutant models showing the functional link between the PGDH3-permitted electron sink and CEF. By linear electron flow (LEF) electrons (e^-^) are directed from photosystem II (PSII) by the reduction of plastoquinone (PQ) to plastoquinol (PQH_2_) over the cytochrome *b*_6_*f* complex (Cyt*b*_6_*f*) and subsequent reduction of plastocyanin (PC) to photosystem I (PSI). Cyt*b*_6_*f* transfers the two H^+^ from PQH_2_ to the thylakoid lumen generating a proton motive force and a ΔpH. Following LEF the electrons can be transferred from PSI to ferredoxin (FD) and be released to the stroma via ferredoxin NADP reductase (FNR) generating NADPH from NADP^+^ and thereby fueling amongst other processes the Calvin Benson Bassham cycle (CBBC). 3-phosphoglycerate (3-PGA) synthesized in the CBBC can be used by PHOSPHOGLYCERATE DEHYDROGENASE3 (PGDH3) as substrate in the first enzymatic step towards serine biosynthesis. PGDH3 generates in this step NADH from NAD^+^ which can then energize the NAD-Malate-Dehydrogenase (NAD-MDH) to convert oxaloacetic acid (OAA) into Malate. As an alternative to LEF, electrons can be cycled back to the PQ-Pool via two CEF routes. In the AA-sensitive route electrons are transferred from FD by the proton gradient regulation 5 (PGR5) PGR5-like photosynthetic phenotype 1 (PGRL1) complex to PQ. The second route is dependent on the NADH dehydrogenase-like (NDH) complex bound via light-harvesting complex I subunits 5 and 6 Lhca5/6 to PSI forming the PSI-NDH supercomplex. This supercomplex cycles back electrons from FD to the PQ-Pool while transporting H^+^ from the stroma to the lumen. The thickness of the black arrows indicates the electron flux from PSI to CBBC, PGR5, and the NDH complex. Red arrows indicate changes to different photosynthetic parameters. Models of LEF and CEF in the WT (**A**) and the impact on both caused by loss of PGDH3 (**B**), PGDH3 in combination with the NDH complex (**C**), and PGDH3 and PGR5 (**D**) are depicted.

The main compensator in response to *PGDH3* loss seems to be CEF, which was increased in *pgdh3* alleles (Fig. 3B, Suppl. Figure 2) and only partly decreased by antimycin A treatments (Fig. 3C). Through the design of *pgdh3pam68l* and *pgdh3pgr5* double mutants, we were able to gain more insights into the relative contribution and importance of either CEF contributor. For clarity, our results are summarized as models comparing the situation on wild-type (Fig. 6A) and mutant plants (Fig. 6B-D). The NDH complex, a Fd-dependent PQ reductase, and a H^+^ pump, lowers the lumenal pH depending on the environmental conditions^29,47^. *pgdh3* mutants exhibit higher NDH activities (Fig. 6B), since we found that loss of NDH activity in *pgdh3pam68l* plants dampens NPQ well below the wild-type level (Fig. 6C). Interestingly, several mutants with defects in CBBC and CBBC-linked reactions exhibit increased NDH activity and high CEF rates^48–52^. Therefore, our finding further solidifies the hypothesis that PGDH3 activity in C_3_ plants is employed to balance CBBC activity by removing 3-PGA buildups and deducting e^-^ from the stromal NADPH pool into NADH. Subsequent NADH oxidation via the NAD-MDH provides a secure e^-^ export route into the cytosol via the malate shuttle.

Interestingly, also increased CEF via the PGR5 involving pathway was described for specifc Arabidopsis mutants^53^. A lack of the PPSB pathway also drives up the activity of the antimycin A-sensitive pathway (Fig. 6B). This was confirmed by low NPQ, low CEF, and an exaggerated fluctuating light stress sensitivity in *pgdh3pgr5* plants compared to respective single mutant and wild-type controls (Fig. 6D). As shown by our OJIP and P700 studies, the molecular foundation for this phenomenon is the extreme PSI acceptor-side limitation found in *pgdh3pgr5* plants (Fig. 4–5). It follows that *pgdh3* mutants utilize PGR5-depedent CEF to divert acceptor-side limitations during photosynthesis induction and light transitions. However, more interestingly our data show that *pgr5* loss-of-function plants depend on an intact PPSB pathway. In an independent study it was shown that overexpression of flavodiiron proteins, which are missing in angiosperms, partially rescues *pgr5* mutants during light stress by providing an additional stromal e^-^ sink^54–56^. Together these results emphasize the so far underestimated importance of PGDH3 and concomitant NADH production to avoid NADP shortages, dangerous PSI acceptor-side limitations, and PSI damage.

In conclusion, the PPSB provides a safe stromal e^-^ sink for C_3_ photosynthesis, which deserves attention as a biotechnological target. The feasibility of engineering plants with improved photosynthesis during light stress needs to be tested with PGDH3 overexpressor lines. In addition, the role of PPSB in monocots and in C_4_ photosynthesis remain open questions awaiting investigation.

## Methods

### Plant growth conditions

Information on all accessions employed in this study in summarized in the accession paragraph below. After 2 days of stratification at 4 °C Col-0 and mutant plants were germinated on soil and grown under 110 µmol photons m^−2^ s^−1^ illumination in a climate chamber with a 16-h/8-h day-night cycle at temperatures of 22 °C/18 °C (light/dark). 3-week-old plants were used for experiments if not stated differently. Fluctuating light treatments were carried out at room temperature under 80 µmol photons m^−2^ s^−1^ for 5 and 1100 µmol photons m^−2^ s^−1^ illumination for 1 min in a 16-h/8-h day-night cycle for 48 h and shifted back to standard growth conditions for 5 days recovery.

### Protein isolation and immunoblotting

Protein isolation and immunoblotting were performed as described previously in Penzler et al*.*, 2022^27^.

### RNA isolation, RNA sequencing, and data analysis

#### RNA extraction and sequencing

We followed our previously established workflow^57^. In short, above ground plant material was harvested from 21-day-old plants, immediately flash frozen in liquid nitrogen, and stored at -80 °C. Four plants per genotype were pooled as a biological replicate. Each genotype was represented by three biological replicates. Frozen plant material was ground to fine powder. RNA was extracted using the RNeasy Plant Mini Kit (Qiagen, Hilden, Germany) with on-column DNAse digestion according to manufacturer’s instructions. The integrity and purity of the RNA was tested spectroscopically and by Bioanalyzer RNA 6000 Nano assay (Agilent, Santa Clara, CA, USA). Library preparation and Illumina paired-end 150 bp sequencing was carried out by BMKGene (Münster, Germany). Raw sequencing files have been deposited on the NCBI short read archive under: PRJNA1099156 (https://dataview.ncbi.nlm.nih.gov/object/PRJNA1099156?reviewer=clvr6lfuhh1jb8fjvtph6kob5s).

#### Read trimming, mapping and DEG calling 

Unless otherwise mentioned, RNAseq data analysis has been conducted on the Galaxy platform^58^ with standard software settings. Raw read files were trimmed with TrimmGalore (https://github.com/FelixKrueger/TrimGalore.com/fenderglass/Flye) and subsequently checked using FastQC (http://www.bioinformatics.babraham.ac.uk/projects/fastqc/). Using the RNAStar software ((Version 2.7.8a)^59^, reads were mapped to the Arabidopsis Tair 10 genome release together with the TAIR 10.52 annotation (Arabidopsis_thaliana.TAIR10.dna.toplevel.fa.gz and Arabidopsis_thaliana.TAIR10.52.gtf downloaded from ensembl.org). Read distribution and gene body coverage of mapped reads was assessed with the RSeQC software package (version 5.0.1)^60^. Aligned reads per gene were subsequently counted by featureCounts (version 2.0.1)^61^ based on the TAIR 10.52 annotation. All log files and outputs of the quality control were combined into one HTML file (Suppl. File 2) using MultiQC (version 1.11)^62^.

Statistical analysis and assessment of differentially expressed genes was performed with limma-voom including EdgeR (version 3.50.1)^63,64^. The limma-voom package was set to low read count filtering to at least 0.5 counts per million in at least 2 samples, TMM read count normalization, and one factor DEG analysis.

#### Data analysis and visualization

Volcano plots and heat maps were produced using the GraphPad Prism software (version 10.2.1; GraphPad Prism Software LLC).

BAM files of mapped reads were imported into the standalone IGV software (Version 2.15.2.11)^65^. The read coverage of the *PGDH3* locus was exported.

To check the top 1000 genes of the RNAseq dataset, a combined read count table was imported into iDEP^66^, read counts were filtered using the integrated EdegR with at least 0.5 counts per million in at least two samples and exporting the gene identifier of the top 1000 diverse genes. This list was used to filter the normalized read count table output of limma-voom. The resulting table was imported into the Perseus software (version 2.0.7.0)^67^. Here the hierarchical clustering heat map was produced using Euclidean row clustering with k-means pre-clustering, complete linkage, and no constraints.

### Photosynthetic measurements

IMAGING- and DUAL-PAM (WALZ, Effeltrich, Germany) measurements were carried out on 3-week-old plants dark-adapted for 15 min prior to each measurement^68,69^. Standard induction curves for NPQ/4 determination were carried out at 110 µmol photons m^−2^ s^−1^. For post-illumination chlorophyll fluorescence transient (PIFT) measurements, 56 µmol photons m^−2^ s^−1^ were applied for 5 min, after which AL was turned off to monitor chlorophyll fluorescence F_t_ for additional 4 min^49^.

PSI and PSII properties under short-term fluctuating light were determined under the same light intensities described for long-term treatments using a DUAL-PAM. Fast chlorophyll a fluorescence induction (OJIP) and plastoquinone pool size were measured following manufacturer instructions. Proton conductivity of the thylakoid membrane (gH^+^), as a proxy for ATPase activity, and ECSt were measured in detached leaves at room temperature at 110 µmol photons m^−2^ s^−1^ using the photosynq MultispeQ V2.0 (PhotosynQ Inc., East Lansing, MI 48,823 USA) system^70^ and the RIDES 2.0 protocol.

## Supplementary Information

Supplementary Information 1.

Supplementary Information 2.

Supplementary Information 3.

## Data Availability

The datasets (raw sequencing) generated during and/or analysed during the current study are available in the NCBI short read archive under: PRJNA1099156 (https://dataview.ncbi.nlm.nih.gov/object/PRJNA1099156?reviewer=clvr6lfuhh1jb8fjvtph6kob5s).

## Declarations

### Accession numbers

The wild-type, T-DNA insertion lines, and mutants explored were from the Arabidopsis thaliana (*A. thaliana*) Columbia 0 (Col-0) accession. T-DNA insertion lines *pgdh3-1* (SM_3_37584; At3g19480); *pgdh3-2* (GK-877F12-026469; At3g19480); *pam68l-1* (SALK_143426; At5g52780)^71^ and point mutation lines *pgr5*^*hope1*^ (At2g05620)^26^; *pgr5*-Cas#1 (At2g05620)^27^; *npq4-1* (At1g44575)^19^ used as single and after crossings as higher order mutants were confirmed by PCR and sequencing respectively.
