# Supplementary material for: Cyclic electron flow compensates loss of PGDH3 and concomitant stromal NADH reduction
Source: Sci Rep. 2024 Nov 26;14:29274. doi: 10.1038/s41598-024-80836-x (PMC11589868; doi:10.1038/s41598-024-80836-x)
Supplement: Supplementary file 1 — Supplementary Information 1. [file 41598_2024_80836_MOESM1_ESM.pdf]

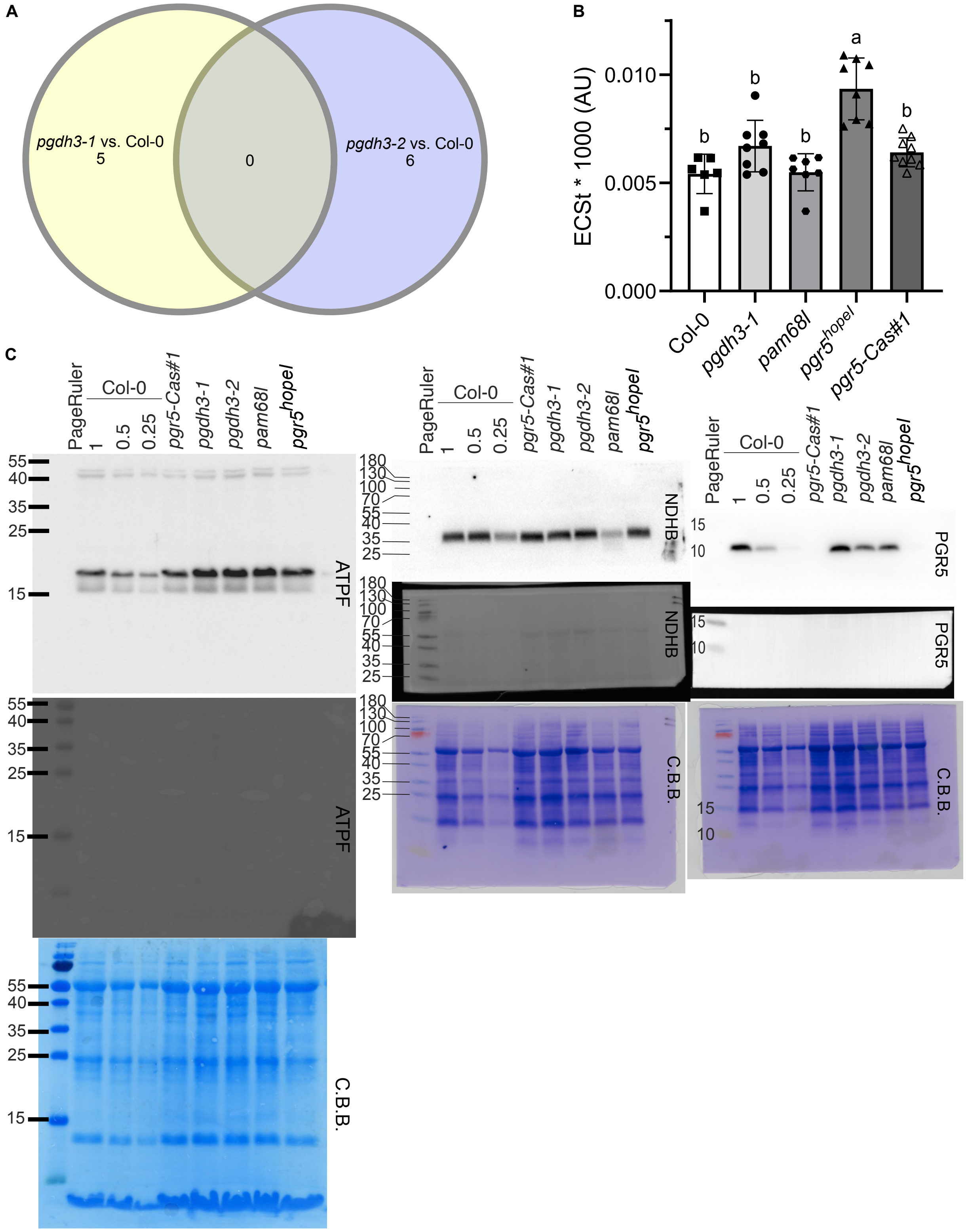

Supplemental Figure 1: Transcriptomics Venn-Diagram of differentially expressed genes reveals no difference between *pgdh3* against Col-0. A Venn-Diagram of *pgdh3-1* and *pgdh3-2* against Col-0 transcriptomic DEGs with FDR < 0.05 and log_2FC = 1. **B** Transthylakoid proton motive force (ECSt) under standard growth conditions in 3-week-old plants. Col-0 (filled squares), *pgdh3-1* (filled circle), *pam68l* (filled hexagon), *pgr5hope1* (filled triangel), *pgr5-Cas#1* (filled triangle). Mean, ± SD, N > 4. **C** full immunoblot membranes from Fig. 2F

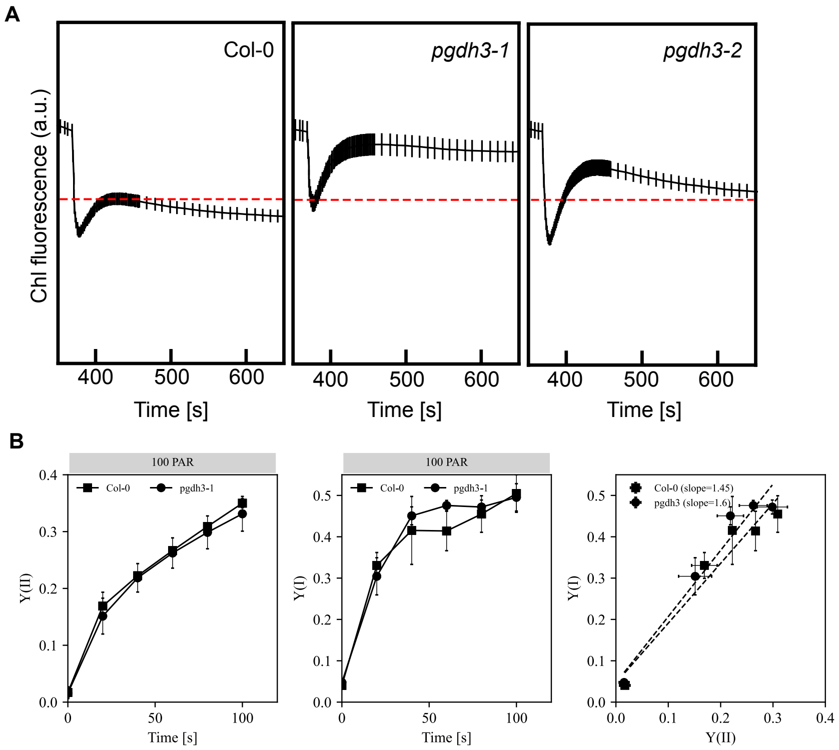

Supplemental Figure 2: Elevated CEF in both *pgdh3* alleles. A PIFT of 3-week-old plants after illumination at 56 PAR of the genotypes Col-0, *pgdh3-1*, and *pgdh3-2*. The dotted red line indicates the Col-0 F_0 peak in PIFT. Mean, ± SEM, N = 9. **B** Yield of electron transport through PSII (Y(II)) and PSI (Y(I)) during the first two minutes of induction at 100 PAR after dark adaptation. The slope of the electron flux through both PSII and PSI are indicated for Col-0 and *pgdh3* alleles. Mean, ± SEM, N = 3.

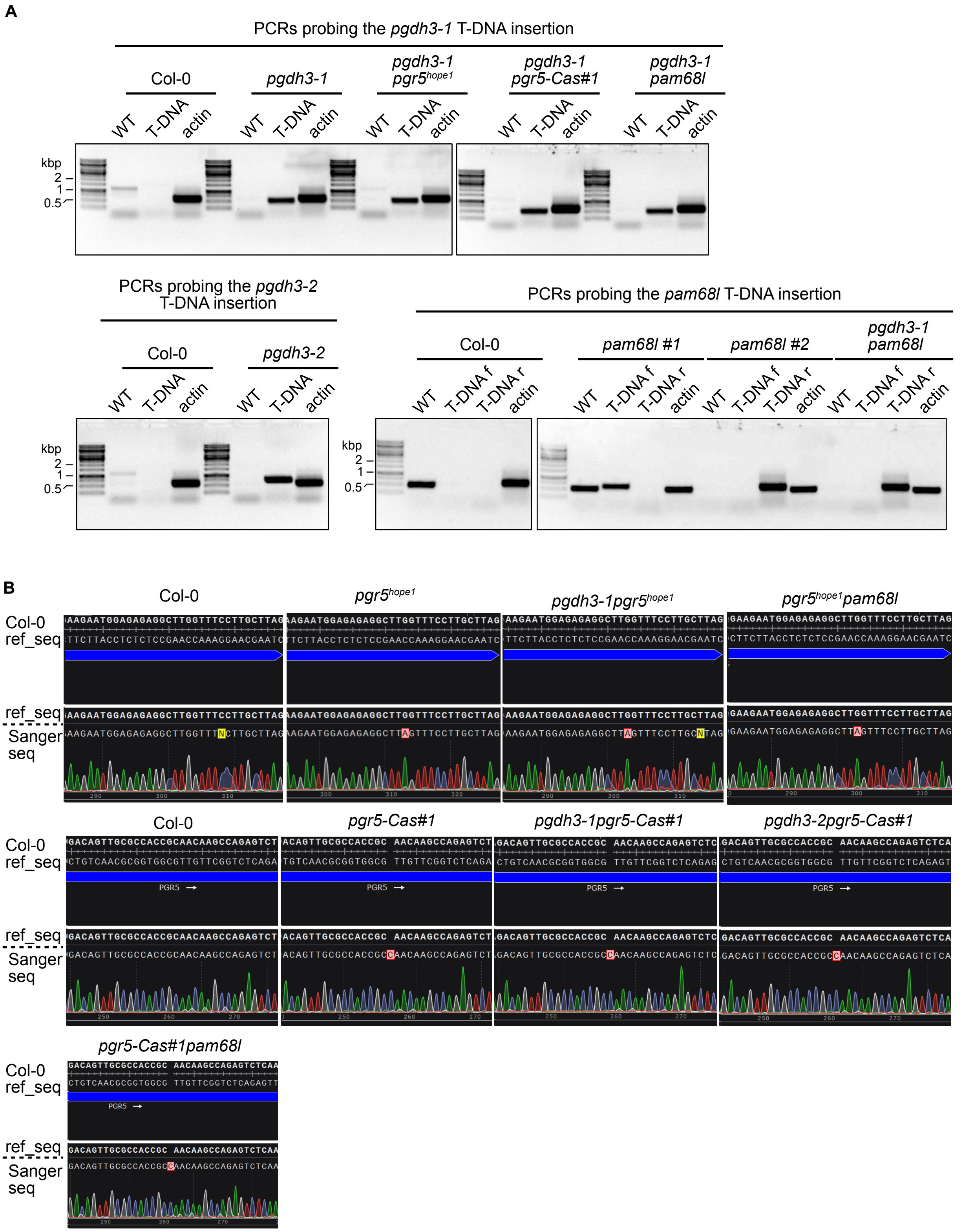

Supplemental Figure 3: Genotyping and re-sequencing of *pgdh3*, *pam68l*, and *pgr5* lines. **A** Genotyping was performed on single and double mutants of either *pgdh3-1*, *pgdh3-2*, and *pam68l*. Primer combinations against WT allele and T-DNA insertions were used as listed in Supplemental Table 1. The allele being genotyped is indicated above the bar. **B** re-sequencing of *pgr5^{hope1}* and *pgr5-Cas#1* single and double mutant lines.

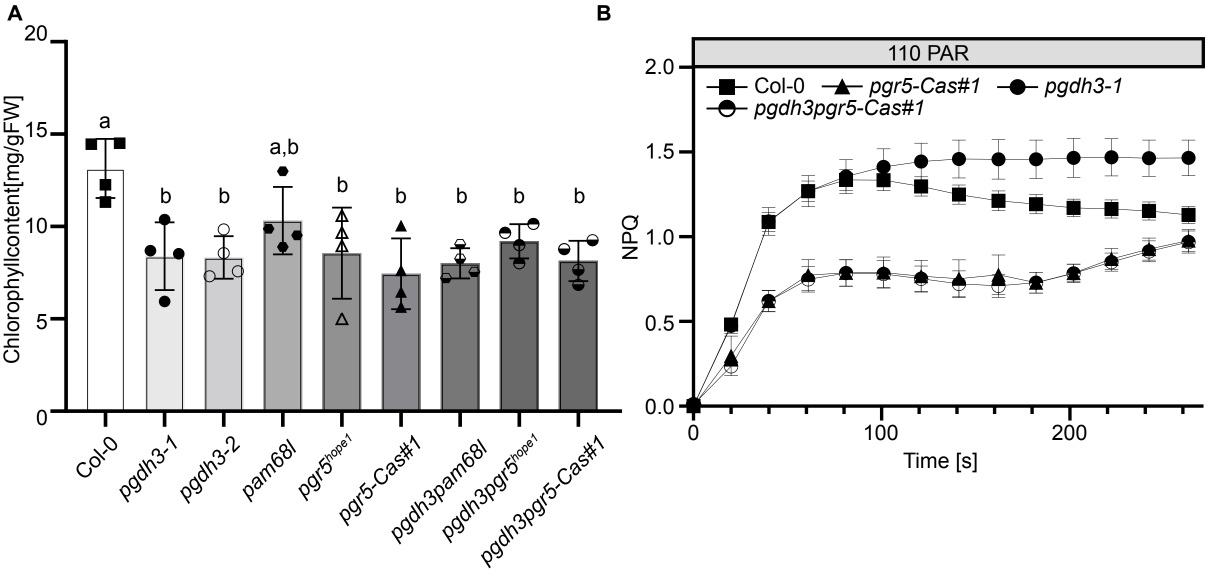

Supplemental Figure 4: Characterization of NPQ in *pgdh3pgr5-Cas#1* and chlorophyll contents in single vs. double mutants. A Chlorophyll contents [mg / g fresh weight (FW)] were calculated for Col-0 (filled squares), *pgdh3*-1 (filled circle), *pgdh3-2* (empty circle), *pam68l* (filled hexagon), *pgr5^{hope1}* (empty triangle), *pgr5-Cas#1* (filled triangle), *pgdh3pam68l* (half-filled hexagon), *pgdh3pgr5-Cas#1* (upper half-filled circle), and *pgdh3pgr5^{hope1}* (lower half-filled circle). Mean, ± SD, N = 4, P < 0.05. **B** Non-Photochemical-Quenching (NPQ) induction curve measured at 110 PAR for Col-0 (filled squares), *pgdh3*-1 (filled circle), *pgr5-Cas#1* (filled triangle), and *pgdh3pgr5^{hope1}* (half-filled circle). Mean, ± SD, N = 6-9

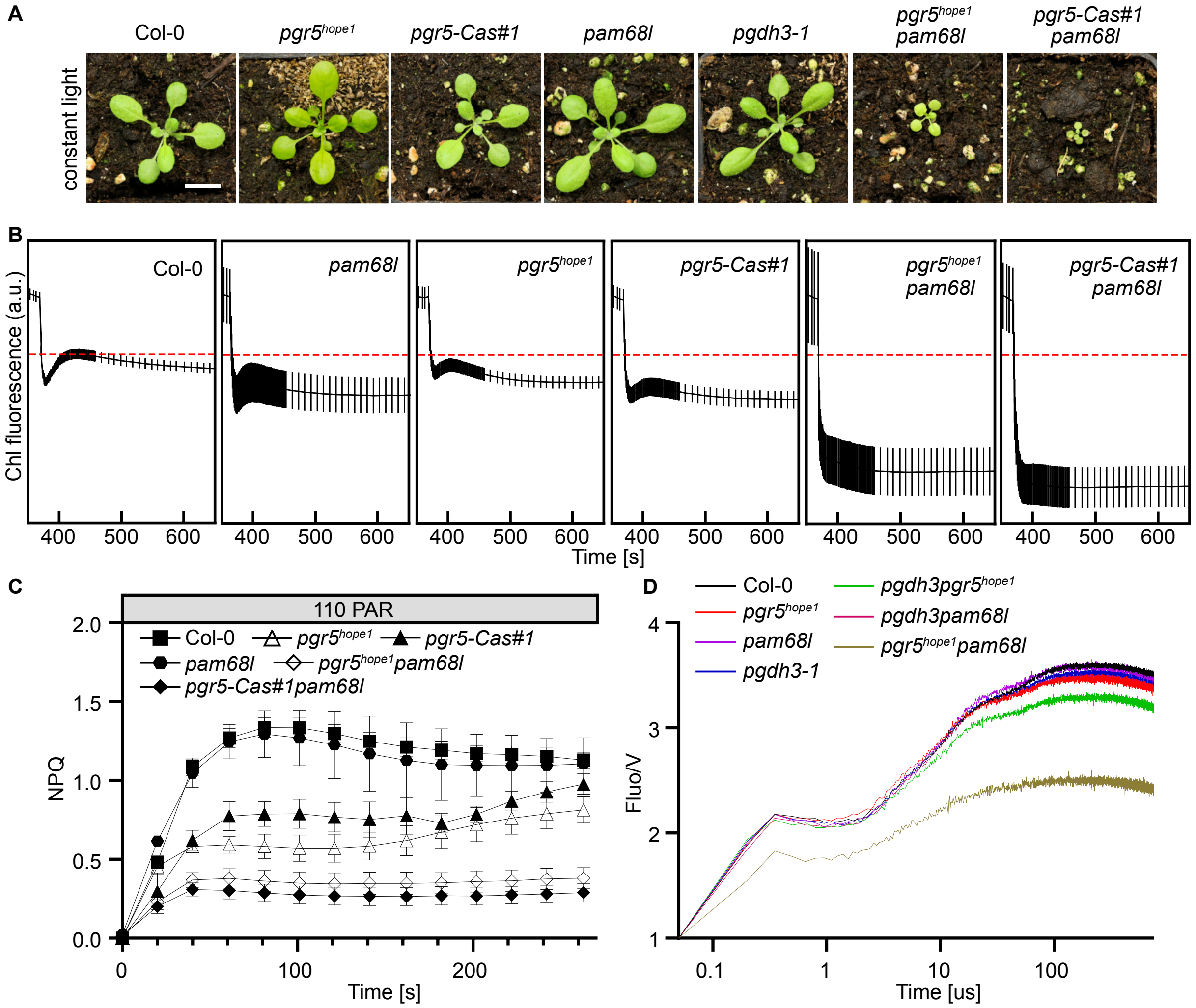

Supplemental Figure 5: Characteristics of *pgr5pam68l* mutants. A 3-week-old plants grown on soil at standard light conditions (110 PAR) in an LD climate chamber. Scale bar = 1 cm. **B** PIFT of 3-week-old plants after illumination at 56 PAR of the genotypes Col-0, *pam68l*, *pgr5*^{hope1}, *pgr5-Cas#1*, *pgdh3pgr5*^{hope1}, and *pgr5-Cas#1pam68l*. The dotted red line indicates the Col-0 F_0 peak in PIFT. Mean, ± SEM, N = 9 **C** Non-Photochemical-Quenching (NPQ) induction curve measured at 110 PAR for Col-0 (filled squares), *pam68l* (filled hexagon), *pgr5*^{hope1} (empty triangle), *pgr5-Cas#1* (filled triangle), *pgr5-Cas#1pam68l* (filled diamond), and *pgr5*^{hope1}*pam68l* (empty diamond). Mean, ± SD, N = 6-9 **D** OJIP curves were taken with the standard Dual-PAM protocol for the different genotypes. Col-0 (black), *pgdh3*-1 (blue), *pam68l* (purple), *pgr5*^{hope1} (red), pgdh3pam68l (pink), *pgdh3pgr5*^{hope1} (green), and *pgr5*^{hope1}*pam68l* (yellow). MEAN, N = 6

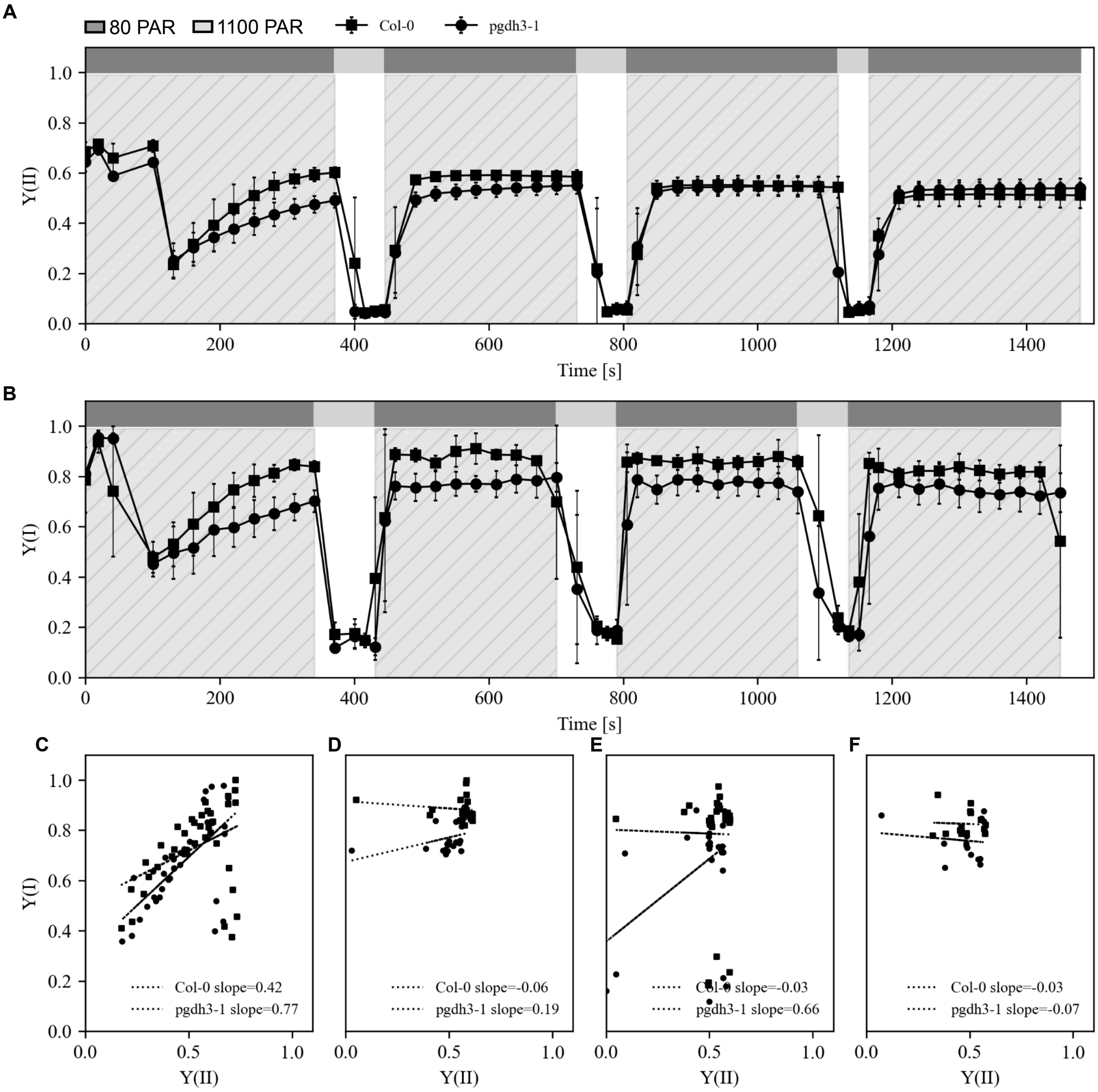

Supplemental Figure 6: Short-term light fluctuations and their effect on CEF in *pgdh3*. A Fluctuating light measurements were carried out with 3-week-old plants to determine effective PSII quantum yield (Y(II)), and **B** photochemical quantum yield of PSI (Y(I)) for the genotypes Col-0 (filled squares) and *pgdh3-1* (filled circle). Data is the same represented in Figure 5. Low light (80 PAR) and high light (1100 PAR) were applied for 5 minutes and 1 minute respectively with 4 low light and 3 high light cycles. Mean, ± SEM, N = 3. **C, D, E, F** Relationship between electron flux through PSII and PSI during the transitions to low light (shaded regions in A and B). The slopes of the linear fits for each genotype following each light transition are indicated.

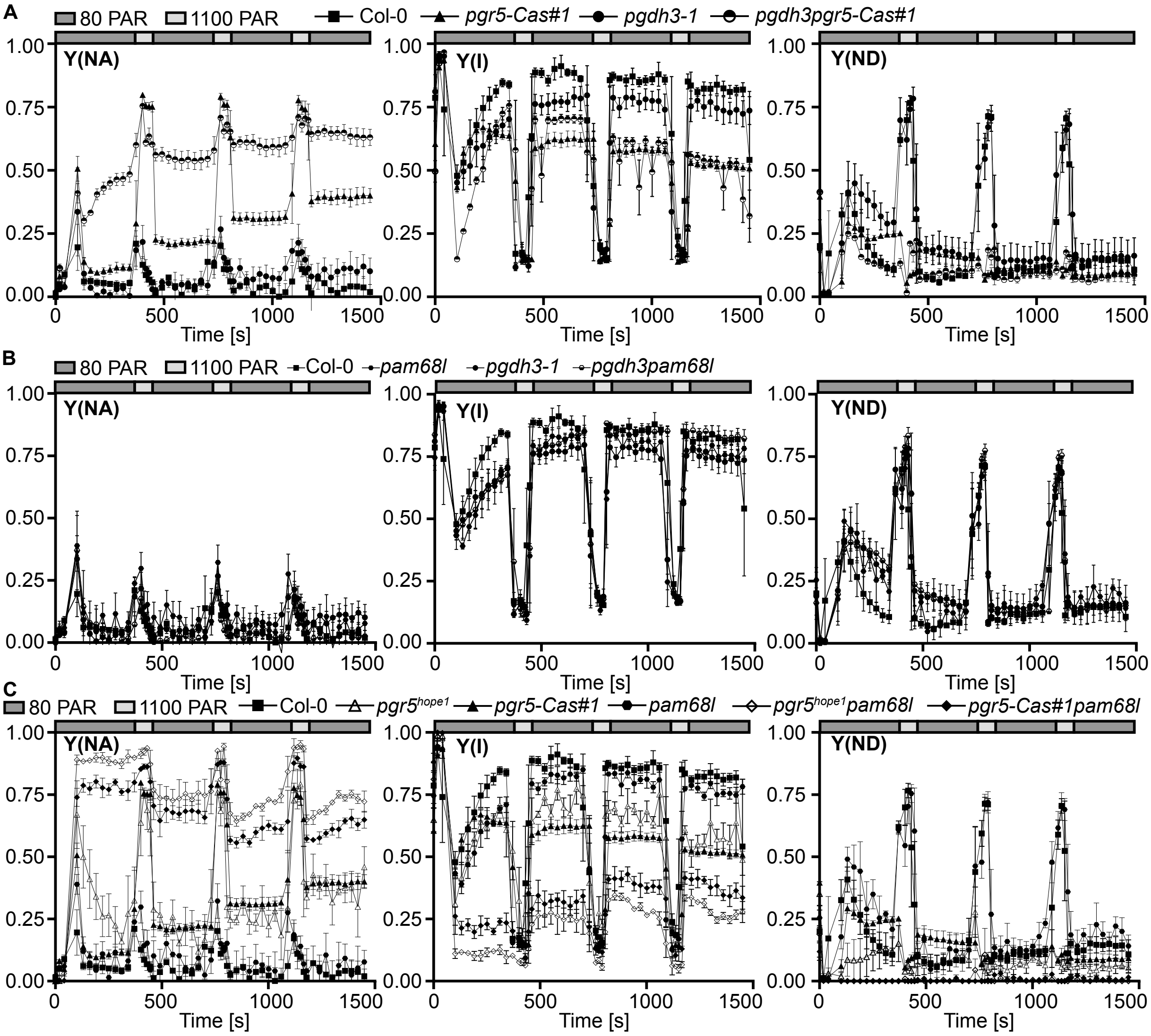

Supplemental Figure 7: Short-term light fluctuations and their effect on PSI-related parameters. A, B, C Fluctuating light measurements were carried out with 3-week-old plants to determine PSI acceptor site limitation (Y(NA)), PSI donor site limitation (Y(ND)), and photochemical quantum yield of PSI (Y(I)) for the genotypes Col-0 (filled squares), *pgdh3-1* (filled circle), *pam68l* (filled hexagon), *pgr5*^{hope1} (empty triangle), *pgr5-Cas#1* (filled triangle), *pgdh3pgr5-Cas#1* (upper half-filled circle), *pgdh3pam68l* (half-filled hexagon), *pgr5-Cas#1pam68l* (filled diamond), and *pgr5*^{hope1}*pam68l* (empty diamond). Low light (80 PAR) and high light (1100 PAR) were applied for 5 minutes and 1 minute respectively with 4 low light and 3 high light cycles. Mean, ± SEM, N = 3

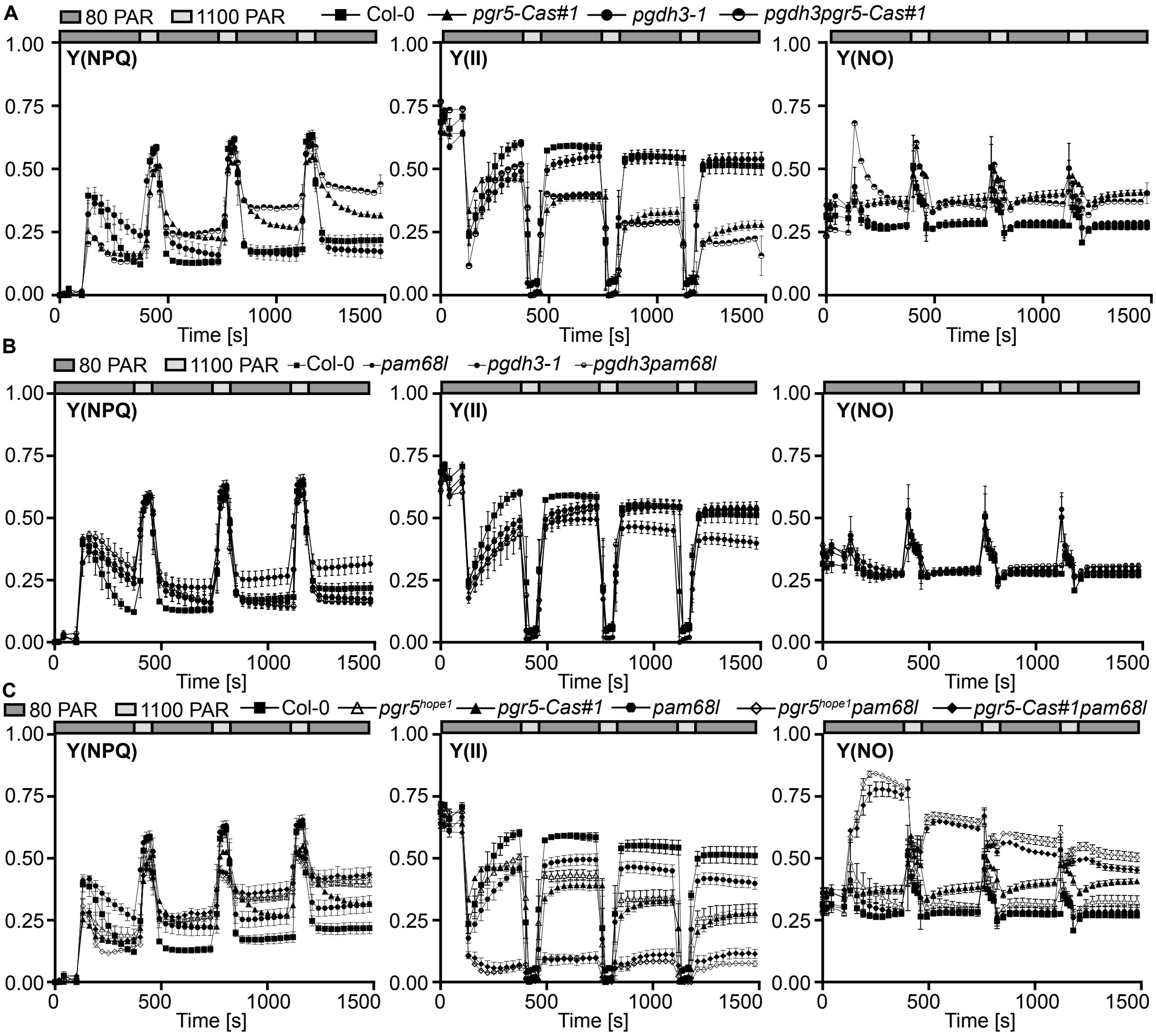

Supplemental Figure 8: Short-term light fluctuations and their effect on PSII-related parameters. A, B, C Fluctuating light measurements were carried out with 3-week-old plants to determine quantum yield of regulated energy dissipation (Y(NPQ)), quantum yield of nonregulated energy dissipation (Y(NO)), and effective PSII quantum yield (Y(II)) for the genotypes Col-0 (filled squares), *pgdh3-1* (filled circle), *pam68l* (filled hexagon), *pgr5^{hope1}* (empty triangle), *pgr5-Cas#1* (filled triangle), *pgdh3pgr5-Cas#1* (upper half-filled circle), *pgdh3pam68l* (half-filled hexagon), *pgr5-Cas#1pam68l* (filled diamond), and *pgr5^{hope1}pam68l* (empty diamond). Low light (80 PAR) and high light (1100 PAR) were applied for 5 minutes and 1 minute respectively with 4 low light and 3 high light cycles. Mean, ± SEM, N = 3

Supplemental Table 1: Oligonucleotide sequences (5' to 3') used in combination for genotyping and sequencing.

Gene	Primer Forward (5' to 3')	Primer Reverse (5' to 3')
pgdh3-1	*pgdh3-1* for:	*pgdh3-1* rev:
WT	ACTTAAACGCGCCTTATCTAATAG	GGCAGATGCAAAGAGATGAAG
T-DNA	ACTTAAACGCGCCTTATCTAATAG	GTTTTGGCCGACACTCCTTACC
pgdh3-2	*pgdh3-2* for:	*pgdh3-2* rev:
WT	ACTTAAACGCGCCTTATCTAATAG	GGCAGATGCAAAGAGATGAAG
T-DNA	CCCATTTGGACGTGAATGT	GGCAGATGCAAAGAGATGAAG
pam68l	*pam68l* for:	*pam68l* rev:
WT	CACAATCCAAAAACCCTATATCC	AGCCAGCTTAAAAGTTTTTATGAG
T-DNA	CACAATCCAAAAACCCTATATCC	ATTTTGCCGATTTCGGAAC
pgr5	*pgr5* for:	*pgr5* rev:
sequencing	CTCTGGTTTCTCCATCCAAAC	CTCCGATCTTAGGGATGCT
